# Supplementary material for: Evaluation of 6-Hydroxydopamine and Rotenone In Vitro Neurotoxicity on Differentiated SH-SY5Y Cells Using Applied Computational Statistics
Source: Int J Mol Sci. 2022 Mar 10;23(6):3009. doi: 10.3390/ijms23063009 (PMC8953223; doi:10.3390/ijms23063009)
Supplement: Supplementary file 1 [file ijms-23-03009-s001.zip › ijms-1613366-supplementary.pdf]

Supplementary figures

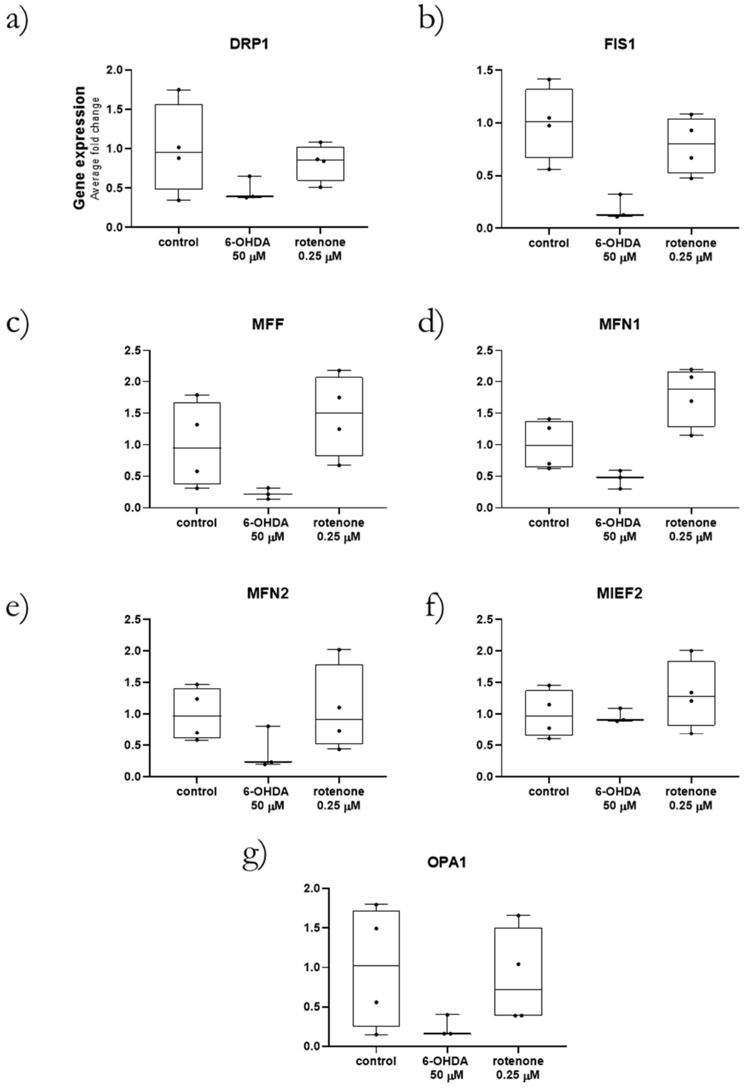

Figure S1. qPCR analysis of genes encoding proteins involved on mitochondrial fusion and fission. Transcript levels of dynamin-related protein 1 (DRP1) (**a**), mitochondrial fission 1 protein (FIS1) (**b**), mitochondrial fission factor (MFF) (**c**), mitofusin 1 (MFN1) (**d**), mitofusin 2 (MFN2) (**e**), mitochondrial elongation factor 2 (MIEF2) (**f**), and optic atrophy 1 protein (OPA1) (**g**) were evaluated after cells were incubated for 24 h with 50 μM 6-OHDA or with 0.25 μM rotenone. Data is represented in boxplots in which each dot represents an independent cell population (n = 3-4). Kruskal-Wallis test (One-way ANOVA on ranks) pairwise (control vs 6-OHDA or control vs rotenone) was used to assess statistical significance.

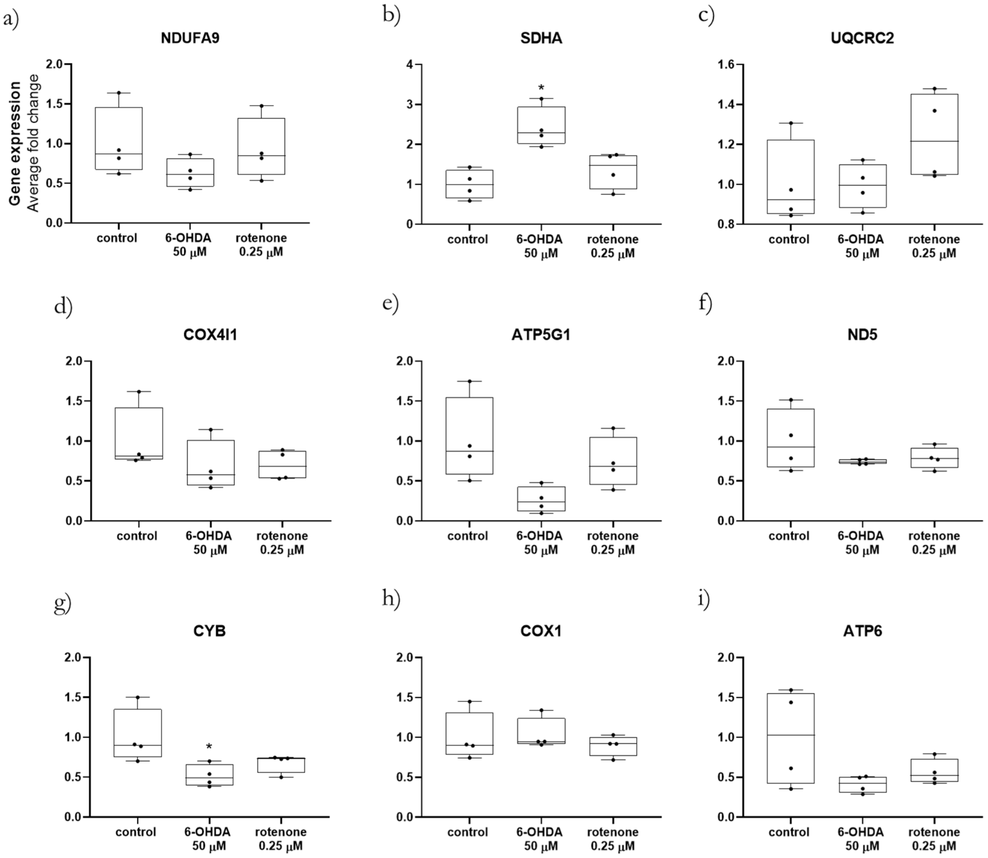

Figure S2. qPCR analysis of electron transport chain complex and ATP synthase subunits encoded by nuclear and mtDNA were altered by both 6-OHDA and rotenone. Nuclear-encoded ETC complex subunits NADH:Ubiquinone oxidoreductase subunit A9 (NDUFA9) (**a**), succinate dehydrogenase complex flavoprotein subunit A (SDHA) (**b**), ubiquinol-cytochrome c reductase core protein 2 (UQCRC2) (**c**), cytochrome c oxidase subunit 4I1 (COX4I1) (**d**), and ATP synthase membrane subunit c locus 1 (ATP5G) (**e**), as well as mtDNA-encoded ETC complex subunits NADH:Ubiquinone oxidoreductase core subunit 5 (ND5) (**f**), cytochrome b (CYB) (**g**), cytochrome c oxidase I (COX1) (**h**), and ATP synthase membrane subunit 6 (ATP6) (**i**) mRNA levels were assessed in cells treated with 50 μM 6-OHDA or with 0.25 μM rotenone for 24 h. Data is represented in boxplots in which each dot represents an independent cell population (n = 4). Kruskal-Wallis test (One-way ANOVA on ranks) pairwise (control vs 6-OHDA or control vs rotenone) was used to assess statistical significance, (*) $p < 0.05$.

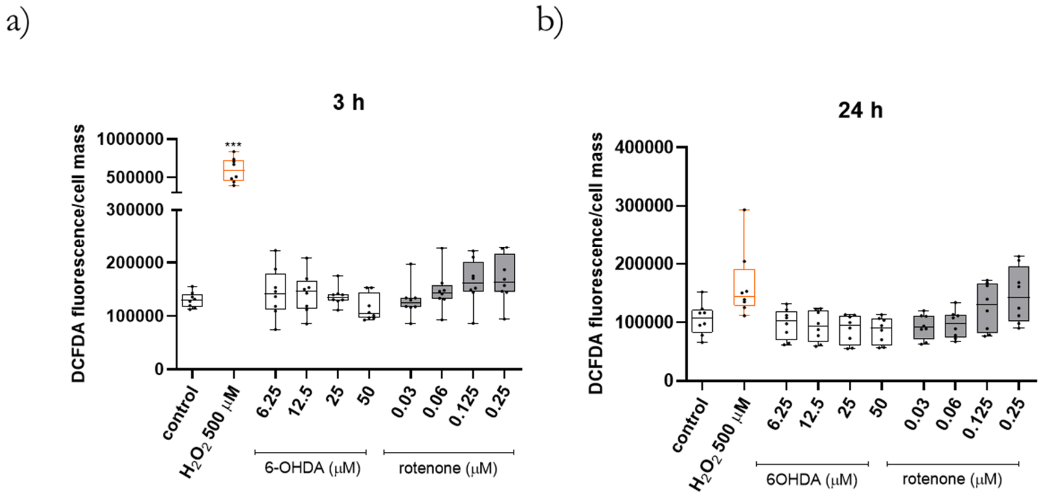

Figure S3. Treatments with both compounds induced an increase in H_2DCFDA oxidation. Cells were treated with non-toxic concentration of 6-OHDA and rotenone for 3 h (**a**) and 24 h (**b**) and consequently ROS production was assessed by measuring the fluorescent levels of H_2DCFDA. Hydrogen peroxide (H_2O_2), 500 μM, was used as positive control. Although no evident alteration in dye fluorescence intensity were found, rotenone-treated cells (0.125 μM and 0.25 μM) displayed an average increase of 26% and 30% (respectively) when incubated for 3 h (**a**) and 19% and 39% increase when treated for 24 h (**b**).

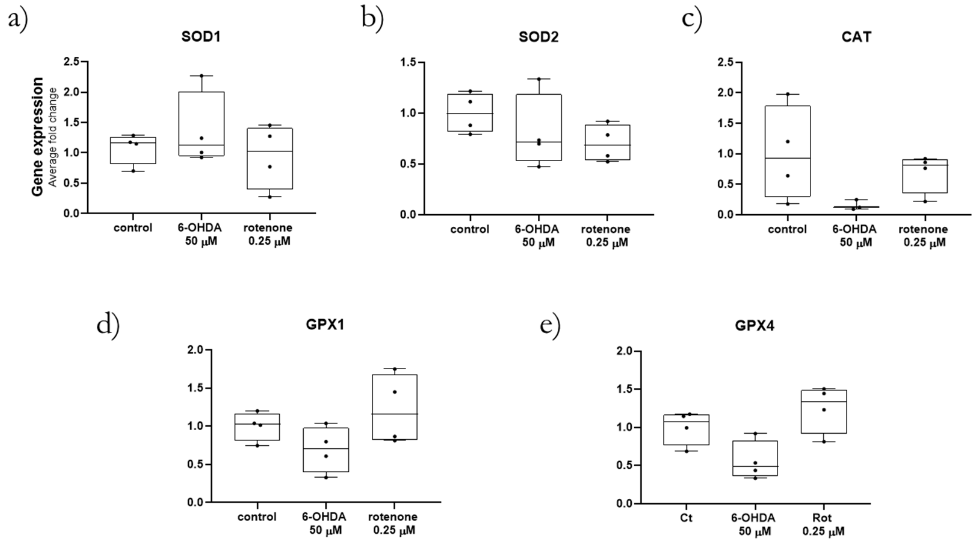

Figure S4. qPCR analysis of selected antioxidant enzymes mRNA levels. mRNA levels of superoxide dismutase 1 (SOD1) (**a**), superoxide dismutase 2 (SOD2) (**b**), catalase (CAT) (**c**), glutathione peroxidase 1 (GPX1) (**d**) and glutathione peroxidase 4 (GPX4) (**e**) were evaluated in cells treated for 24 h with 50 μM 6-OHDA and with 0.25 μM rotenone. Data is represented in boxplots in which each dot represents an independent cell population (n = 3-4). Kruskal-Wallis test (One-way ANOVA on ranks) pairwise (control vs 6-OHDA or control vs rotenone) was used to assess statistical significance.
